# Dietary Practice and Nutritional Status of Tuberculosis Patients in Pokhara: A Cross Sectional Study

**DOI:** 10.3389/fnut.2018.00063

**Published:** 2018-08-16

**Authors:** Lal M. Gurung, Laxman D. Bhatt, Isha Karmacharya, Dipendra K. Yadav

**Affiliations:** Pokhara University, School of Health and Allied Sciences, Lekhnath, Nepal

**Keywords:** tuberculosis, nutrition, diet, TB patients, Nepal

## Abstract

**Background:** Undernutrition increases the risk of progression from Tuberculosis (TB) infection to active TB disease and further leads to weight loss. Proper diet and nutrition play significant roles in treating TB patients. Active TB needs high energy requirement. The main aim of this study is to assess the dietary intake and nutritional status of TB patients in Pokhara city of Nepal.

**Methods:** A cross-sectional descriptive study was carried out among 133 TB patients taking anti-tubercular drug. Data were collected using sequential sampling method. Data were collected from 4th October to 7th November, 2016.

**Results:** This study revealed that about one-fifth of TB patients did not consume sufficient amount of calories as per RDA. More than one-third of patients were underweight during the time of registration and this is reduced to 21.8 percent in the present situation. Mean BMI was 20.99 kg/m^2^ (SD ± 5.81). Similarly, the mean BMI among Pulmonary TB (PTB) is 19.82 and 22.52 kg/m^2^ in Extra PTB. Working conditions and food intake frequency were significantly associated with calorie intake. This study found that the amount of calories, food frequency per day, types of TB, and nutritional status during registration were found to be associated with recent nutritional status. The statistical difference between mean BMI at registration and recent BMI and mean weight at registration and recent weight.

**Conclusion:** Nutritional status has improved comparatively from the time of registration to the time of study. Proper nutritional counseling should be given to TB patients along with nutritional support to severely malnourished patients, and nutritional assessment of TB patients should be done periodically.

## Introduction

Tuberculosis (TB) is caused by *Mycobacterium tuberculosis* which often affect the lungs, although it can spread to other organs in the body (1). TB is the second deadliest disease worldwide caused by a single infectious agent (1). Globally, in 2014, 9.6 million people were estimated to have fallen ill with TB; among them, 3.2 million were women, 5.4 million were men, and 1.0 million were children. Of the 9.6 million TB cases, in 2014, 12% of them were HIV positive. Now, TB ranks along with HIV as a leading cause of death worldwide (2). However, TB is completely curable through a short course of chemotherapy (DOTS), which has been recognized as a highly cost-efficient and effective strategy (3).

TB spreads from person to person through the air (4). There are two kinds of TB infections: latent and active. In latent TB, the bacteria remain inactive and do not show any typical signs and symptoms of TB. While they are not contagious, they can become active at any time. In active TB, the bacteria show signs and symptoms of TB and are contagious to others (1). Symptoms of active pulmonary TB are coughing, chest pain, fever, night sweats, weight loss, fatigue, and sometimes, the coughing up of blood (5). Active TB, like other infectious diseases, requires high energy consumption i.e., 20–30% extra energy of recommended daily allowance (RDA). Undernutrition increases the risk of progression from TB infection to an active TB disease. Food insecurity and poor nutritional status in the population are important contributors to the global burden of the TB disease (6).

Both malnutrition and TB are of considerable magnitude in most of the underdeveloped regions of the world. Nutritional status is significantly lower in patients having active TB than others (7). Therefore, the present study focused on the dietary intake and nutritional status of TB patients in Pokhara, Nepal.

## Methods

This was a cross-sectional descriptive study conducted among TB patients taking anti-tubercular drug in Pokhara, Nepal, from 4th October to 7th November, 2016. The total number of TB patients in Pokhara were 203 in FY 2072/73. The sample size was determined by assuming the prevalence of malnutrition in TB patients to be 51% (8), 95% confidence limit, and 5% marginal error, and the sample size was 133.

List of total 12 Directly Observed Treatment Short Course (DOTS) centers in Pokhara was obtained from District Public Health Office (DPHO), Kaski, and arranged from numbers one to twelve. A sequential sampling technique was used. Of them, the required sample size was met at the 11th DOTS center. Patients who visited the DOTS center during the data collection period were included in the study.

The structured questionnaire was developed referencing STEP wise approach to Surveillance (STEPS) survey, Nepal (9, 10), and was translated into the Nepali language. The questionnaire was pre-tested on 10% of the total sample in Tanahun, Nepal. Primary data collection was performed through face-to-face interviews. Tools for the data collection were a structured questionnaire, checklist (patient treatment card), bathroom scale weighing machine for weight measurement, stature meter for height measurement, and locally available cups, plates, and glasses for measuring the amount of food consumed.

Data were entered into EpiData software and analyzed by using SPSS 20 version software. Univariate and bivariate analyses were performed for data analysis. The size of dishes used to measure the amount of food was of 250 ml, which was equivalent to a standard cup size. Through this reference, the amount of food was converted into a standard serving size, and the daily energy intake was calculated by Nutrition facts 0.9.5.0 version and Food tables (11).

The study protocol was reviewed and approved by the Ethical Review Committee of Pokhara University Research Centre and permission was taken from DPHO Kaski. Informed verbal consent was obtained from all the participants prior to data collection, and data confidentiality was maintained.

## Results

Out of 133 participants, the majority (33.1%) of them were between 20 and 30 years of age, with the mean age being 35.23 years (SD ± 15.05). More than half (60.9%) of them were males. Most were predominantly upper caste by ethnicity and Hindu by religion (Table 1).

**Table 1:** Socio-demographic characteristics of TB patients (*n* = 133).

	**Characteristics**	***n***	**%**
Age group	10–20	18	13.5
	20–30	44	33.1
	30–40	27	20.3
	40–50	26	19.5
	50–60	7	5.3
	more than 60	11	8.3
Mean = 35.23 years, SD = 15.05, Min^m^ = 14, Max^m^ = 82			
Sex	Male	81	60.9
	Female	52	39.1
Family Type	Single	73	54.9
	Joint	60	45.1
Family Size	≤5 members	95	71.4
	>5 members	38	28.6
Mean = 4.92, SD = 2.27, Min^m^ = 1, Max^m^ = 14			
Marital status	Unmarried	45	33.9
	Married	78	58.6
	Separated	2	1.5
	Widow	8	6.0
Ethnicity	Dalit Caste	20	15.0
	Disadvantaged Janajati Caste	21	15.8
	Disadvantaged Non Dalit Terai Caste	3	2.3
	Advantaged Janajati Caste	44	33.1
	Upper Caste	45	33.8
Religion	Hindu	115	86.4
	Buddhist	13	9.8
	Muslim	1	0.8
	Christian	1	0.8
	Others	3	2.2
Occupation	Agriculture	37	27.9
	Employment	25	18.8
	Household activity	24	18.0
	Business	16	12.0
	Student	16	12.0
	Labor	12	9.0
	Others(Trekking)	3	2.3
Education	Illiterate	15	11.3
	No-formal education	22	16.5
	Lower secondary	28	21.0
	Secondary	32	24.1
	Higher secondary	36	27.1

Information related to diseases and health service utilization among TB patients is given in Table 2. More than half of the patients have Pulmonary TB. 57.9% TB patients started medication in less than 1 month after diagnosis.

**Table 2:** Disease and health services utilization related information.

**Characteristics**	***n***	**%**
Types TB (*n* = 133)		
Pulmonary TB	73	54.9
Extra PTB	60	45.1
Disease duration (*n* = 133)		
< 6 months	101	75.9
≥6 months	32	24.1
Median = 4, IQR = 3, Min^m^ = 0, Max^m^ = 13
Occurrence of complication (*n* = 133)		
Yes	43	32.3
No	90	67.7
Co-morbid diseases (*n* = 29)		
Diabetes	9	31
Arthritis	4	13.9
Hypo-tension	4	13.9
Kidney disease	3	10.3
Hypertension	2	6.9
Gastritis	2	6.9
Thyroid	1	3.4
HIV/AIDs	1	3.4
Others	3	10.3
Treatment CAT (*n* = 133)		
CAT I	119	89.5
CAT II	14	10.5
Start to take medicine after illness (*n* = 133)		
≤ 1 month	77	57.9
>1 month	56	42.1
Mean = 0.97, SD = 1.67
Phase of medication (*n* = 133)		
Intensive phase	37	27.8
Continuous phase	96	72.2
Duration of missing medication (*n* = 15)		
Not defaulted	14	93.3
Defaulted	1	6.7
Median = 6 days, IQR = 11, Min^m^ = 1, Max^m^ = 99

Information related to food and its consumption is given in Table 3. The majority (78.2%) of the participants consumed a sufficient amount of calories, whereas 21.8% did not. The mean was 3239.39 (SD ± 1352.47).

**Table 3:** Food related information.

**Characteristics**		***n***	**%**
Frequency of food intake in a day (*n* = 133)	≤4 times >4 times	47 86	35.3 64.7
	Median = 4, IQR = 1, Min^m^ = 2, Max^m^ = 6		
Meal not prepared at house consumption per week (*n* = 66)	≤2 days >2 days	26 40	39.4 60.6
	Median = 2, IQR = 4		
Protein rich food^*^	Pulses	123	92.5
	Meat	121	91.0
	Egg	107	80.4
	Milk and milk product	104	78.2
	Fish	57	42.9
Protective food^*^	Vegetables	130	97.7
	Fruits	125	93.9
Calorie intake per day (*n* = 133)	Not sufficient Sufficient	29 104	21.8 78.2
	Mean = 3239.39 Kcal, SD = 1352.47, Min^m^ = 339Kcal, Max^m^ = 7626.75 Kcal		

* *based on multiple response analysis*.

The nutritional status among TB patients is given in Table 4. Mean weight and height were 51.98 kg and 1.57 m, respectively. Relatively, a higher percent were underweight (21.8%) than overweight (17.3%).

**Table 4:** Nutritional Status of TB patients.

**Characteristics**		***n***	**%**
Recent nutritional status (*n* = 133)	Underweight Normal Over weight	29 81 23	21.8 60.9 17.3
	Mean BMI = 20.99 k.g/m^2^, SD = 5.81, Min^m^ = 13.699 kg/m^2^, Max^m^ = 32.8999 kg/m^2^		
**FURTHER CLASSIFICATION OF NUTRITIONAL STATUS**			
Undernutrition (*n* = 29)	Severe thinness Moderate thinness Mild thinness	6 4 19	20.7 13.8 65.5
Over weight (*n* = 23)	Pre-obese Obese class I	19 4	82.6 17.4
**BASIS OF CALORIE INTAKE**			
Not sufficient calorie intake (*n* = 29)	Malnutrition Normal Over weight	9 13 7	31 44.9 24.1
Sufficient calorie intake (*n* = 104)	Malnutrition Normal Over weight	20 68 16	19.2 65.4 15.4

Factors which are associated with calorie intake are shown in Table 5. Working conditions and food intake frequency were significantly associated with calorie intake. Socio-demographic factors and disease-related factors were not found to be associated with calorie intake.

**Table 5:** Factors associated with calorie intake.

**Characteristics**		**Not sufficient**	**Sufficient**	***p*-value**	**UOR (95% CI)**
**CALORIE INTAKE**					
Sex	Male	19 (23.5%)	62 (76.5%)	0.565	1.29 (0.54–3.04)
	Female	10 (19.2%)	42 (80.8%)		
Age	≤30	13 (21.7%)	47(78.3%)	0.97	0.98 (0.43–2.25)
	>30	16 (21.9%)	57 (78.1%)		
Ethnicity	Dalit/Disadvantaged group	12 (27.3%)	32 (72.7%)	0.28	1.58 (0.68–3.7)
	Upper/Advantaged group	17 (19.1%)	71 (80.9%)		
Religion	Hindu	28 (23.7%)	90 (76.3%)	0.24#	4.35 (0.54–34.6)
	Other than Hindu	1 (6.7%)	14 (93.3%)		
Working condition	Working	33 (35.5%)	60 (64.5%)	0.007^*^	3.85 (1.36–10.7)
	Not working	5 (12.5%)	35 (87.5%)		
Education	Illiterate	4 (26.7%)	11 (73.3%)	0.88#	–
	Primary level	7 (20.6%)	27 (79.4%)		
	Higher than primary	18 (21.4%)	66 (78.6%)		
Food frequency per day	≤4 times	15 (31.9%)	32 (68.1%)	0.037^*^	2.40 (1.04–5.57)
	>4 times	14 (16.3%)	72 (83.7%)		
Types of TB	Pulmonary TB	15 (20.5%)	58 (79.5%)	0.699	0.85 (0.37–1.93)
	Extra PTB	14 (23.3%)	46 (76.7%)		
Treatment category	CAT I	24 (20.2%)	95 (79.8%)	0.18	0.45 (0.14–1.48)
	CAT II	5 (35.7%)	9 (64.3%)		
Duration of start medication after illness	≤1 month	17 (22.1%)	60 (77.9%)	0.929	1.03 (0.45–2.39)
	>1 month	12 (21.4%)	14 (78.6%)		
Co-morbid condition	Yes	8 (27.6%)	21 (72.4%)	0.39	1.50 (0.58–3.87)
	No	21 (20.2%)	83 (79.8%)		

* *Statistically significant p-value from bivariate analysis, #p-value from likelihood ratio*.

Factors that are associated with recent nutritional status of TB patients are given in Table 6. Food frequency, TB types, calorie intake, and nutritional status at the time of registration were significantly associated with the recent nutritional status of the participants.

**Table 6:** Factors associated with recent nutritional status.

**Characteristics**		**Normal**	**Malnutrition**	***p*-value**	**UOR (95% CI)**
**RECENT NUTRITIONAL STATUS**					
Sex	Male	49 (60.5%)	32 (39.5%)	0.904	0.96 (0.46–1.95)
	Female	32 (61.5%)	20 (38.5%)		
Family size	≤5 members	57 (60.0%)	38 (40.0%)	0.73	0.87 (0.43–1.9)
	>5 members	24 (63.2%)	14 (36.8%)		
Food frequency per day	≤4 times	21 (44.7%)	26 (53.3%)	0.005^*^	0.34 (0.16–0.73)
	> 4 times	60 (69.8%)	26 (30.2%)		
Types of TB	Pulmonary TB	32 (43.8%)	41 (56.2%)	0.025^*^	0.45 (0.22–0.91)
	Extra PTB	38 (63.3%)	22 (36.7%)		
Calorie intake	Not sufficient	13 (44.8%)	16 (55.2%)	0.045^*^	0.43 (0.186–0.992)
	Sufficient	68 (65.4%)	36 (34.6%)		
Nutrition at registration	Normal	61 (87.1%)	9 (12.9%)	<0.001^*^	14.57 (6.05–35.06)
	Malnutrition	20 (31.7%)	43 (68.3%)		

* *Statistically significant p-value from bivariate analysis*.

Statistical differences between mean BMI at registration and recent BMI (MD −1.04; 95% CI −1.05 to −1.39; *p* < 0.001) and mean weight at registration and recent weight (MD −1.52; 95% CI −1.004 to −1.003; *p* < 0.001) are shown in Table 7.

**Table 7:** Mean difference between BMI and Weight at registration and recent BMI and Weight.

	***p*-value**	**Mean difference**	**95% CI**
BMI	<0.001^*^	−1.04	−1.05 to −1.39
Weight	<0.001^*^	−1.52	−1.004 to −1.003

* Statistically significant p-value based on t-test

## Discussion

This study revealed that about one-fifth of TB patients did not consume sufficient amounts of calories as per the RDA. The study found that 36.1% were underweight and 11.35% were overweight during the time of registration. In contrast, studies conducted in other countries found that 51% in Ghana (8), more than 85% in India (12), 70.6% in Brazil (13), 57% in Malawi (14), and more than 58% in Tanzania (15) were underweight during the time of registration. It might be due to the difference in sampling size and study setting. This study found that male patients were more underweight than females. In contrast, a study conducted in India found that females were more underweight than males during the time of registration (12). However, nutritional status improved by 15.3% in patients who had completed two months of medication. Other studies showed that nutritional status was improved by 11% (8) and 13.5% (12) at the end of the treatment.

The prevalence of co-morbidity was 21.85%, where diabetes was the most prevalent (31%) followed by hypotension and arthritis. A study done in Tanzania showed that prevalence of diabetes among PTB was 6.7% (16). In India, annual cases of TB increased to 46% among people with diabetes (17).

This study found that the amount of calorie, food frequency per day, types of TB, and nutritional status during registration was found to be associated with recent nutritional status. However, socio-demographic factors were not associated with nutritional status, whereas a study in Tanzania found that sex was significantly associated with nutritional status (18). In this study, the majority (86.5%) of patients belonged to the Hindu religion. A study conducted in immigrant Asians from South London revealed Hindu Asians were found to have a significantly increased risk for TB compared with Muslims (19).

## Conclusions

This study revealed that about one-fifth of TB patients did not consume the sufficient amount of calories as per the RDA. The Mean calorie intake was 3239.39 (SD ± 1352.47). More than one-third of the patients were underweight at the time of registration, but this is reduced to 21.6% in the present situation. The mean BMI was 20.99 kg/m^2^ (SD ± 5.81): 20.56 kg/m^2^ in males and 21.67 kg/m^2^ in females. Similarly, mean BMI among PTB was 19.82 and 22.52 kg/m^2^ in Extra PTB. The prevalence of co-morbidity was 21.85%, where diabetes was the most prevalent (31%). Working conditions and food intake frequency were significantly associated with calorie intake. This study found that the amount of calories, food frequency per day, types of TB, and nutritional status during registration were found to be associated with the recent nutritional status. There was statistical differences between mean BMI at registration and after treatment and mean weight at registration and after treatment. It was increased after treatment. The nutritional status has improved comparatively from the time of registration to the present situation. Proper nutritional counseling along with nutritional support should be given to severely malnourished TB patients, and nutritional assessment of TB patients should be done periodically.

## Ethics statement

Ethical clearance was obtained from the institutional review committee of Pokhara University at the beginning of the study. Detailed information was available in Nepali to all participants. Confidentiality was maintained and the information was used for research purposes only. Each participants participated voluntarily in this study. Written consent was taken from all the participants before the data collection. Participants were informed about their BMI status. In case of an abnormal range, educational counseling was given regarding a balanced diet and they were referred to a nutritionist for the further services.

## Author contributions

LG was the principle investigator for the study. She compiled all contributor tasks, generated the topic and reviewed the related literature. LG and LB facilitated data collection. DY designed the study methodology and overall analysis and helped write the results section. LG, LB, and IK drafted the manuscript. DY reviewed the manuscript.

### Conflict of interest statement

The authors declare that the research was conducted in the absence of any commercial or financial relationships that could be construed as a potential conflict of interest.

## References

[1] McIntosh J Tuberculosis: causes, symptoms and treatments. Medical News Today (2015).

[2] WHO. Global Tuberculosis Report 2015. 25462184

[3] WHO What is DOTS (Directly Observed Chemotherapy, Short Course). SEARO (2016).

[4] WHO Tuberculosis. Geneva: World Health Organization (2015).

[5] Sinclair D Abba K Grobler L Sudarsanam TD Nutritional supplements for people being treated for active tuberculosis. Cochrane Database Syst Rev. (2011) 9:CD006086 10.1002/14651858.CD006086 22071828

[6] WHO Guideline: Nutritional Care and Support for Patients With Tuberculosis. Geneva: World Health Organization (2013). 24624480

[7] Gupta KB Gupta R Atreja A Verma M Vishvkarma S. Tuberculosis and nutrition. Lung India (2009) 26:9. 10.4103/0970-2113.45198 20165588 PMC2813110

[8] Dodor E. Evaluation of nutritional status of new tuberculosis patients at the effia-nkwanta regional hospital. Ghana Med. J. (2008) 42:22–28. 18560556 PMC2423338

[9] Kumar Aryal K Neupane S Mehata S Vaidya A Singh S Paulin F Non Communicable Diseases Risk Factors: STEPS Survey Nepal 2013. (2013).

[10] Lamsal A Assessment of Food Intake by Children Attending Montessori School of Pokhara Valley Faculty of Health Science. Lekhnath: Pokhara University (2015).

[11] Park K Park's Textbook of Preventive and Social Medicine, 19th Edn. Jabalpur: Banarsidas Bhanot Publishers (2007).

[12] Bhargava A Chatterjee M Jain Y Chatterjee B Kataria A Bhargava M. Nutritional status of adult patients with pulmonary tuberculosis in rural central India and its association with mortality. PLoS ONE (2013) 8:e77979. 10.1371/journal.pone.0077979 24205052 PMC3812022

[13] Bacelo AC Ramalho A Brasil PE Cople-Rodrigues Cdos S Georg I Paiva E Nutritional supplementation is a necessary complement to dietary counseling among tuberculosis and tuberculosis-HIV patients. PLoS ONE (2015) 10:e0134785 10.1371/journal.pone.0134785 26313258 PMC4551799

[14] Zachariah R Spielmann M Harries A Salaniponi F. Moderate to severe malnutrition in patients with tuberculosis is a risk factor associated with early death. Trans R Soc Trop Med Hyg. (2002) 96:291–4. 12174782 10.1016/s0035-9203(02)90103-3

[15] Kennedy N Ramsay A Uiso L Gutmann J Ngowi F Gillespie S. Nutritional status and weight gain in patients with pulmonary tuberculosis in Tanzania. Trans R Soc Trop Med Hyg. (1996) 90:162–6. 8761578 10.1016/s0035-9203(96)90123-6

[16] Mugusi F Swai A Alberti K McLarty D. Increased prevalence of diabetes mellitus in patients with pulmonary tuberculosis in Tanzania. Tubercle (1990) 71:271–6. 2267680 10.1016/0041-3879(90)90040-f

[17] Dye C Bourdin Trunz B Lonnroth K Roglic G Williams BG. Nutrition, diabetes and tuberculosis in the epidemiological transition. PLoS ONE (2011) 6:e21161. 10.1371/journal.pone.0021161 21712992 PMC3119681

[18] PrayGod G Range N Faurholt-Jepsen D Jeremiah K Faurholt-Jepsen M Aabye MG. Sex, smoking, and socioeconomic status are associated with body composition among tuberculosis patients in a deuterium dilution cross-sectional study in Mwanza, Tanzania. J Nutr. (2013) 143:735–41. 10.3945/jn.112.168997 23514764

[19] Strachan DP Powell KJ Thaker A Millard FJ Maxwell JD. Vegetarian diet as a risk factor for tuberculosis in immigrant south London Asians. Thorax (1995) 50:175–80. 7701458 10.1136/thx.50.2.175 PMC473919

